# Metabolomics analysis of quality components metabolism during the growth process of pepino (*Solanum muricatum*) fruit

**DOI:** 10.1080/15592324.2023.2283363

**Published:** 2023-11-17

**Authors:** Wenwen Zhao, Xuemei Sun, Lihui Wang, Zhu Sun, Huajing Zhang, Qiwen Zhong, Shipeng Yang

**Affiliations:** a Qinghai Key Laboratory of Vegetable Genetics and Physiology, Agriculture and Forestry Sciences Institute of Qinghai University, Xining, China; b College of Agriculture and Animal Husbandry, Qinghai University, Xining, China; c Key Laboratory of Qinghai-Tibet Plateau Biotechnology Ministry of Education, Qinghai University, Xining, China; d College of Life Sciences, Northwest A&F University, Yangling, China

**Keywords:** Pepino (*Solanum muricatum*), metabolism, transcriptome, fruit quality, maturation

## Abstract

Pepino (*Solanum muricatum*), a horticultural crop that has experienced significant growth in the highlands of China over the past two decades, is widely embraced by consumers due to its distinctive taste and nutritional advantages. This study focused on the cultivar ‘Qingcanxiang’ of pepino grown on the Qinghai-Tibetan Plateau was analyzed using UPLC-QTOF-MS and RNA-seq transcriptome sequencing. Fruit samples were collected at three distinct stages of development, and the results of the metabolomics and transcriptomics were compared and correlated. The study’s findings indicate that the ‘Qingcanxiang’ fruit contained a total of 187 metabolites, comprising 12 distinct categories of compounds, including amino acids and their derivatives, organic acids, sugars and alcohols, phenols and phenolic acids. Of these metabolites, 94 were identified as differential. Significant variations in nutrient composition were observed across the three growth stages of the fruit. Specifically, the stage spanning from the growth to the maturation was identified as the critical stages for nutrient accumulation and flavor development. Transcriptome sequencing analysis revealed a set of highly associated genes between aspartate and quinic acid, namely *SIR2*, *IRAK4*, *RP-L29*, and *CCNH*. These genes are potentially involved in the regulation of both amino acid and phenolic acid synthesis. Through the application of metabolomics and transcriptomics, this investigation elucidates the alterations in metabolites and the underlying molecular regulatory mechanisms of pepino fruits during three growth stages. The findings furnish a theoretical foundation for the evaluation of nutritional quality and the enhancement of breeding strategies for pepino.

## Introduction

Pepino (*Solanum muricatum*), originating from the northern foothills of the Andes Mountains in South America, it is a perennial plant of the Solanaceae family and genus Solanaceae with a berry fruit.^1^ Different varieties of pepinos exhibit varying shapes and colors, encompassing both round and elongated forms. The prevalent cultivars of pepino feature skins that are predominantly golden or creamy white, adorned with purple stripes.^2^ The ‘Qingcanxiang’ cultivar of pepino displays an oblong shape with a pale yellow-colored fruit. It is characterized by its aromatic profile, sweetness, and juiciness. Pepino, as an emerging fruit crop, derives its prominent commercial value from its nutritional composition and content. The introduction of this crop to mainland China occurred in the 1980s, and its development has exhibited significant acceleration within the country over the past two decades. Currently, pepino is considered an important economic fruit and is extensively cultivated, particularly in regions such as the Qinghai-Tibet Plateau.^3,4^ The plateau’s unique high-altitude climate, characterized by cool temperatures, significant day-night temperature variations, and abundant sunlight, contributes to the exceptional quality and high yield of pepino produced in these areas.^5^

The production and content of metabolites constitute an important component of fruit quality, as they can directly or indirectly impact the color, flavor, aroma, and texture of fruits. Additionally, they reflect the physiological status of fruits during their growth, development, and storage processes.^6^ Researches have been conducted to analyze the nutritional composition and metabolic compounds present in pepino. These researches have identified various organic acids, alkaloids, vitamins, sugars, alcohols, phenolic acids, and other metabolic substances in pepino.^7^ Furthermore, the focus has been placed on the flavor and volatile aroma compounds of pepino. As scientific research progressed, the insufficiency of traditional methods became apparent, leading to the gradual emergence of metabolomic and transcriptomic techniques starting in the 1990s. Metabolomics can identify key metabolites in plant developmental metabolic networks and metabolic regulatory mechanisms.^8^ By utilizing metabolite profiling and metabolomics, valuable information regarding the biochemical and nutritional components of fruits can be obtained,^9^ making metabolomics highly applicable in studies related to fruit development. Transcriptomics is the field of molecular biology that studies the expression of genes by cells or organisms under specific conditions, usually by measuring the presence and amount of RNA molecules. Using a combination of metabolome and transcriptome, we are able to link changes in metabolomic profiles and gene expression as we explore the relationship between genes and metabolites to decipher this complex coordination.^10^ Consequently, many studies using metabolomics and transcriptomics in parallel have been performed to monitor development. Over the past few decades, the combination of metabolomic analysis and transcriptomic data has been widely used to identify plant metabolites and elucidate metabolic pathways that can be used to explain many physiological reactions.^11^ For example, combined transcriptional and metabolomic analyses have been used to map spatial and temporal regulatory networks during tomato growth and development, to provide insights into the evolution and fruit development of chayote (*Sechium edule*) and to reveal the mechanism of fruit color formation in peppers, etc.^12–14^

Several studies have conducted investigations into the variations in nutritional composition and quality of pepino across different varieties, employing a combined transcriptomic and metabolomic methodology.^10^ However, the mechanisms underlying the accumulation of metabolites in pepino remain unclear. The process of fruit development, maturation and senescence is intricate, and the progression of these developmental changes arises from the interplay between metabolites and genes.^15–17^ The accumulation of metabolites during fruit development follows certain patterns and is influenced by various factors such as fruit developmental stages, fruit varieties, and growth conditions. The examination of the physiological and molecular mechanisms underlying fruit development and ripening not only expands our comprehension of biological growth processes, but also furnishes valuable insights for cultivation practices, such as the efficient regulation of fruit quality and the augmentation of nutritional content during cultivation. Additionally, the analysis of pertinent molecular mechanisms can expedite the development of superior new varieties. However, the understanding of metabolome dynamics and the gene regulatory networks associated with the developmental maturation of pepino is currently lacking. Consequently, the objective of this study was to investigate the accumulation patterns of metabolites during the growth stages of pepino and explore their potential mechanisms in transcriptional regulation.

## Materials and methods

### Plant materials

**Figure 1.**
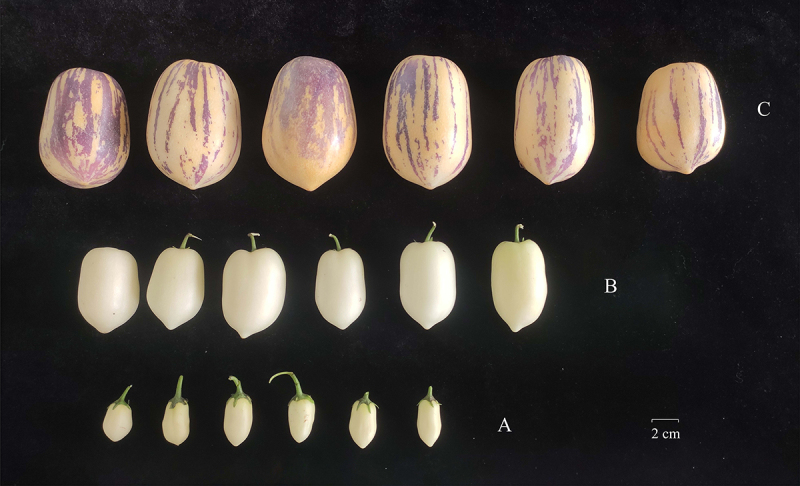
Fruit phenotypes of ‘Qingcanxiang’ pepino at different stages. A initial stage, b: growth stage, c: maturation stage.

The pepino cultivar chosen for this experiment was ‘Qingcanxiang’, planted at the Institute of Horticulture, Qinghai Academy of Agriculture Forestry Science (36°38‘N, 101°55‘E, altitude 2200 m). Fruits of ‘Qingcanxiang’ were collected at the initial stage (90 days after flowering), growth stage (110 days after flowering) and maturation stage (130 days after flowering) of fruit development, and divided into three groups A, B and C in turn (Figure 1), with six repeats in each group. The collected fruits were immediately frozen in liquid nitrogen and subsequently stored at −80°C for preservation.

### Sample preparation

The ‘Qingcanxiang’ fruit samples were retrieved from the −80°C freezer, cut into small pieces, mixed, weighed 50 mg, and placed in 2 mL centrifuge tubes. Added 400 μL of pre-cooled 80% methanol for metabolite extraction for a total of 6 biological replicates, 3 technical replicates per biological replicate. Steel beads were added to a 2 mL centrifuge tube and ground in a mill at −20°C for 60 s at 50 Hz until the material was well ground. The samples were then sonicated in a water bath at 4°C for 30 min and incubated at −20°C for 30 min, followed by centrifugation at 12,000 × g for 10 min. 300 µL of supernatant was transferred to another clean centrifuge tube. After centrifugation at 12,000 × g for another 10 min, transfer 150 µL of supernatant to the injection vial with a liner tube and tighten the cap. All samples were mixed to make QC samples, and a total of 4 QC samples were made.

### Metabolomic and transcriptomic assays

#### UPLC-Q-TOF/Ms

The UPLC-Q-TOF/MS characteristics and the protocol were the following: ACQUITY UPLC HSS T3 column (2.1 mm × 100 mm, 1.8 µm); mobile phase: (A) 0.1% formic acid aqueous solution; (B) 0.1% formic acid acetonitrile solution; gradient elution (0–3 min, 98–95%A; 3–7 min, 95–90% A; 7–8 min, 90–89% A; 8–20 min, 89–80% A, 20–23 min, 80–98% A, allowed re-equilibration of the column to initial conditions); the column temperature was 30°C; flow rate was 0.4 mL/min; Xevo G2-XS type UPLC-Q/TOF-MS liquid mass spectrometer, electrospray ionization source negative ion mode (ESI-), capillary voltage was set to 2000 V, cone well voltage was set to 40 V, ion source temperature was maintained at 100°C, desolvent temperature was 400°C, collision energy was 10 V, desolventizing nitrogen flow rate was 800 L/h, and cone hole backblast nitrogen flow rate was 100 L/h. The desolventizing gas was nitrogen, and the collision gas was argon. The scanning range was 50–1500 m/z.

#### RNA extraction and sequencing

Total RNA was extracted from the fruit samples of pepino using the RNAprep Pure Plant Plus Kit (TIANGEN, Beijing, China). RNA extraction and detection: (1) Agarose gel electrophoresis: analysis of RNA integrity and the presence of DNA contamination. (2) NanoPhotometer spectrophotometer: detection of RNA purity (OD260/280OD260/230). (3) Qubit 2.0 fluorometer: high accuracy measurement of RNA concentration. (4) Agilent 2100 Bioanalyzer: accurate detection of RNA integrity. After extraction of total RNA and digestion of DNA with DNase, eukaryotic mRNA was enriched with magnetic beads with Oligo (dT); the mRNA was broken into short fragments by adding interruption reagent, used as a template to synthesize a single-stranded cDNA with a six-base random primer, and purified with a double-stranded cDNA, which was finally amplified by Polymerase Chain Reaction amplification RNA was extracted and cDNA libraries were constructed.

### Metabolomic data analysis

The MS-DIAL software^18^ was used to deconvolve, peak extract, compare, baseline calibrate, inverse fold and normalize the metabolome downstream data. The positive and negative ion mass spectrometry data were downloaded from the MoNA (MassBank of North America, 2007 Free Software Foundation) database.^19^ The identified metabolites were compared. With default values of: [M-H]^−^, [M-H]^+^ forms of the added ions.

### Statistical analysis

#### Data standardisation

Statistical analysis of data on the MetaboAnalyst 5.0 online platform.^20^ Following are the methods used to normalize metabolism data in MetaboAnalyst 5.0: (1) Element missing values > 80% were removed from the analysis, and the missing values were replaced with 1/5 of the minimum positive value of the corresponding variable. (2) The median absolute deviation (MAD) of the data was used for data filtering. (3) Statistical normalization (i.e., normalizing samples, transforming data, and scaling data): Statistics normalization was performed by normalizing samples by median, transforming data by log (base 10), and scaling data by log. “Auto-scaling” (mean centered and divided by standard deviation).

#### Differentially Accumulated Metabolites (DAMs) screening

The metabolites in the fruit samples of pepino from three different time periods were classified by compound types and a heatmap was generated in R (version 4.2.0). Analysis of variance (ANOVA, *p* < 0.05) was used to analyze the significance of differences in the metabolites of pepino from the three growth periods, and orthogonal partial least squares discriminant analysis (OPLS-DA) was used to screen for differential metabolites in samples from the three periods, with VIP ≥ 1.0 as the screening condition and *p* <.05.

The differentially expressed metabolites obtained were subjected to species enrichment analysis using circos. The metabolites of pepino fruits from three periods were analyzed separately for differences between two groups (A vs B, B vs C and A vs C), the Fold Change (FC) and *p*value were obtained, their log_2_ (FC) and -log_10_ (*p*value) values were calculated, a threshold (|log_2_ (FC)| ≥ 1, p <.05) was set and volcano plots were generated. All of the above analyses were done in the Omicshare (https://www.omicshare.com/).

A network analysis was conducted on the differential metabolites. The R ingraph package was utilized to generate the network, with a Pearson correlation coefficient employed to measure the correlation between nodes. A correlation coefficient threshold of > 0.8 and a correlation coefficient *p* value threshold of 0.05 were applied, and any isolated nodes were eliminated.

#### Metabolome and transcriptome association analysis

The metabolome and transcriptome of pepino fruits were analyzed for association, the differential metabolites with high VIP values in the fruits were selected the Pearson correlation coefficients of metabolites and genes were calculated using the cor function in R, the genes with Pearson correlation coefficients greater than 0.985 with the selected metabolites were displayed, and the network map between the differential metabolites and related genes was drawn using Cytoscape 3.7.1 software to explore the key genes.

## Results

### Analysis of metabolites in pepino fruit

**Figure 2.**
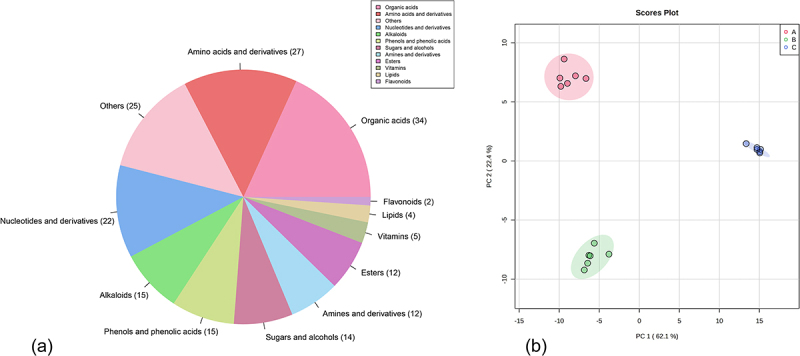
Comprehensive quality control of metabolite analysis. (a) classification and proportion of total 187 metabolites detected in pepino fruits; (b) metabolite principal component analysis (PCA) among samples of pepino fruit at different stages.

In this study, the metabolite profile and relative contents of ‘Qingcanxiang’ fruits were determined at three different growth periods. A total of 187 metabolites were identified (Table S1), of which 106 were in the positive ion mode and 81 in the negative ion mode, after elimination of identified duplicates. The 187 metabolites identified were normalized in the MetaboAnalyst 5.0 platform (Figure S1). These metabolites can be grouped into 12 classes in total (Figure 2a), of which organic acids were the most numerous (34), followed by amino acids and derivatives (27), nucleotides and their derivatives (22), alkaloids (15), phenols and phenolic acids (15), sugars and alcohols (14), amines and derivatives (12), esters (12), vitamins (5), lipids (4), flavonoids (2) and other metabolites (25). The correlation coefficients between replicates in all three growth periods of pepino fruits were above 0.83, and the correlation coefficients between replicates within the fruit sample groups at both the initial and growth stages were above 0.98 (Figure S2), demonstrating that the samples of pepino fruits at the three developmental stages were well replicated and the metabolomic data obtained from the identification were reliable. The unsupervised PCA model was used to analyze the metabolomic data of pepino fruits in the three stages (Figure 2b). The results demonstrated excellent intra-group reproducibility and clear discrimination among the three groups, showcasing notable differences in metabolic profiles.

### Differentially Accumulated Metabolites (DAMs) analysis

**Figure 3.**
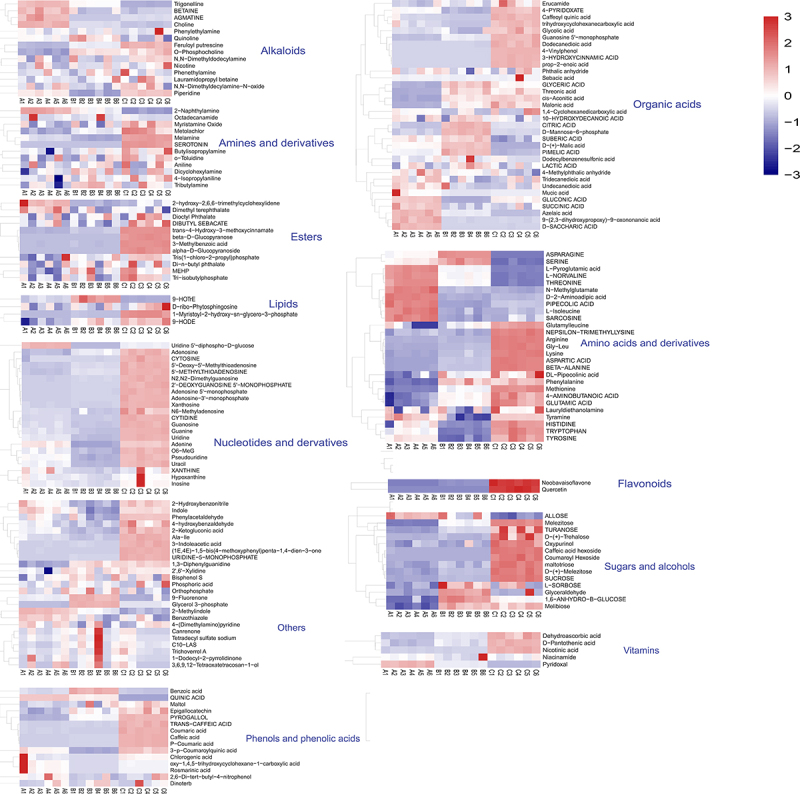
Heatmap analysis of the abundance of metabolites in pepino fruit in three different stages.

**Figure 4.**
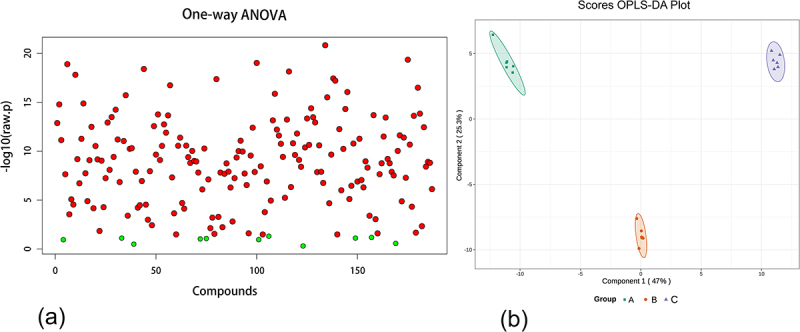
Screening of differential metabolites. (a) ANOVA analysis of differential metabolites; (b) orthogonal partial least squares discriminant analysis (OPLS-DA) for metabolite screening of pepino from three different developmental stages.

**Figure 5.**
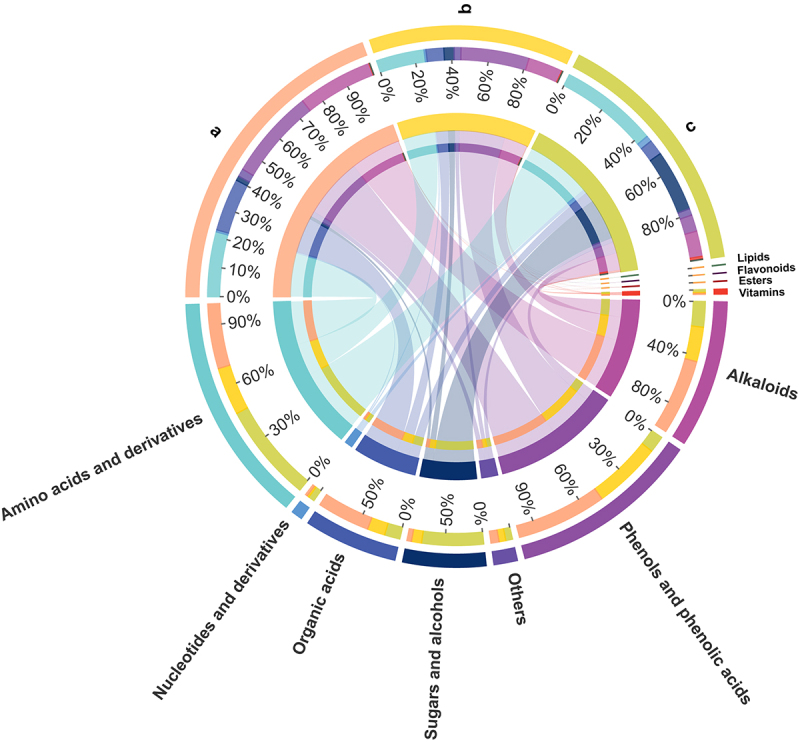
Circos plots demonstrating the abundance of each major metabolite in pepino samples from three different developmental stages normalized based on metabolite abundance.

The heatmap (Figure 3) illustrated the changes in metabolite profiles during fruit development, revealing a gradual increase in the majority of metabolite levels throughout the developmental stages of pepino fruits. Particularly during the transition from the fruit growth stage to maturation stage, nucleotides and derivatives, flavonoids, most amino acids and derivatives, as well as a majority of sugars and alcohols, were significantly upregulated. However, there were also some metabolites that showed significant downregulation, such as organic acids. One hundred and seventy-six metabolites out of a total of one hundred and eighty-seven metabolites identified were found to be differential by ANOVA analysis. In combination with orthogonal partial least squares discriminant analysis (OPLS-DA), 94 differential metabolites were screened for VIP values ≥ 1 and *p* < 0.05 in this model (Figure 4b), and these 94 metabolites were included in the 176 differential metabolites screened by ANOVA analysis. The 94 differential metabolites obtained from the filtering were classified (Table S2) into 11 main groups, of which amino acids and derivatives contributed the most, with 21.28%, followed by nucleotides and their derivatives, with 14.89%.Other abundant metabolites identified included organic acids (13.83%), sugars and alcohols (11.70%), phenols and phenolic acids (7.45%), alkaloids (6.38%), vitamins and esters (both 4.26%), and flavonoids and lipids (2.13%). These compounds had represented the major components implicated in pepino fruit growth and development. The relative content of metabolites in pepino was ranked, and the outermost ring of the circos plot displayed the relative content of metabolites from the most to the least abundant group (Figure 5), with amino acids and derivatives being the most prevalent among the 11 categories of differential metabolites, followed by phenols and phenolic acids. In contrast, organic acids, sugars and alcohols showed diverse profiles with lower abundances. The inner rings of the circos plot revealed significant metabolite variations across the initial, growth and maturation stages. Amino acids and derivatives increased from 30.66% to 46.62%, mirroring nucleotides and derivatives. Vitamins rose from 17.1% to 61.35%. The increasing amino acids and derivatives and vitamins constitute key nutrients in pepino fruits. Interestingly, organic acids declined markedly from 61.37% to 17.92% between the initial and maturation stages, as did phenols and phenolic acids from 51.52% to 9.49%. Conversely, sugars and alcohols progressively increased from 5.81% to 80.87% over maturation. The decreasing organic acids and phenolics coupled with accumulating sugars and alcohols suggest dynamic regulation of flavor-related pathways during pepino fruit development It is presumed that organic acids, phenols and phenolic acids, as well as sugars and alcohols, are strongly associated with the formation of flavor in pepino fruit^21,22^ and that the stage from growth to maturation is the main period of flavor formation in pepino fruits.

**Figure 6.**
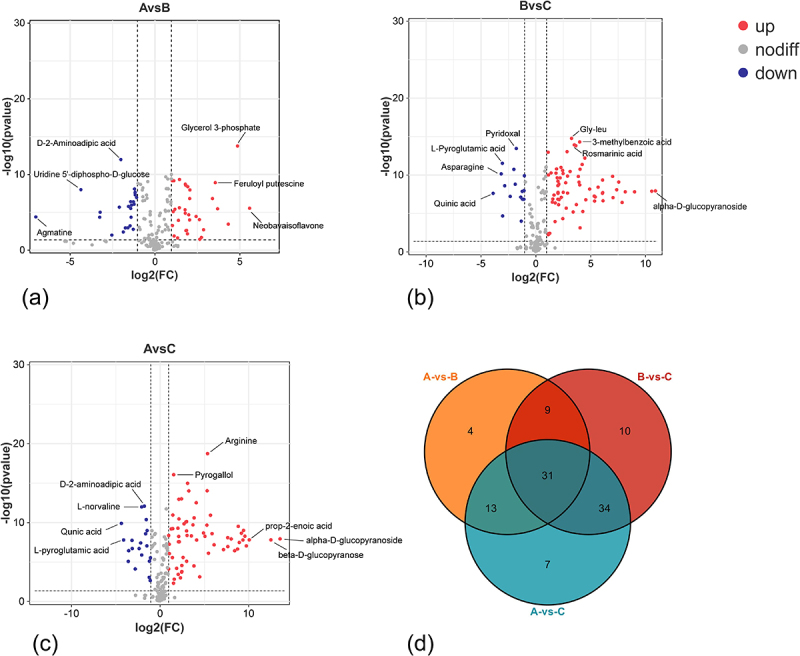
Screening of differential metabolites in pepino fruits during growth. (a) volcano plot of differential metabolite at a vs B stage; (b) volcano plot of differential metabolite at B vs C stage; (c) volcano plot of differential metabolite at a vs C stage; (d) venn plot of differential metabolites among three stages.

The volcano plot exhibited the accumulation of metabolites in pepino fruit across distinct groups. A total of 57 metabolites demonstrated differential abundance between group A vs B. Among these metabolites, 33 exhibited up-regulation, while 24 exhibited down-regulation during the growth stage in comparison to the initial stage. (Figure 6a, Table S3). In the maturation stage of fruit compared to the growth stage, 68 metabolites exhibited up-regulated, while 16 metabolites showed down-regulated, resulting in a total of 84 significantly differentially regulated metabolites (Figure 6b, Table S4). A total of 85 differentially regulated metabolites were identified in the group A vs C, with 65 showing up-regulated and 20 exhibiting down-regulated during fruit maturation compared to the initial stage (Figure 6c, Table S5). It could be seen that most of the nutrients had accumulated gradually as the fruits of pepino grew to maturity, and that the critical period for nutrient accumulation in fruit development was from the growth stage to maturation stage. These results were in agreement with the results of PCA and OPLS-DA analyses and demonstrated that these metabolites had differed significantly among the three developmental stages of pepino fruits. Additional analysis of the differential accumulated metabolites common to the three developmental stages of pepino fruits. As could be seen from the Venn plot (Figure 6d), there had been 31 common differential metabolites in these groups, with amino acids and derivatives, organic acids and phenolic acids accounting for the greatest proportion, with a total of 29.03% for the three classes of compounds. Accordingly, it could be assumed that, these three kinds of compounds had been the main contributors to the DAMs in pepino fruit at different developmental stages. The nutritional quality of pepino fruit is closely related to the amino acids and derivatives, organic acids and some of the main secondary metabolites such as sugars and alcohols. These compounds had played a very important role in maintaining the quality and nutritional value of the fruit.

**Figure 7.**
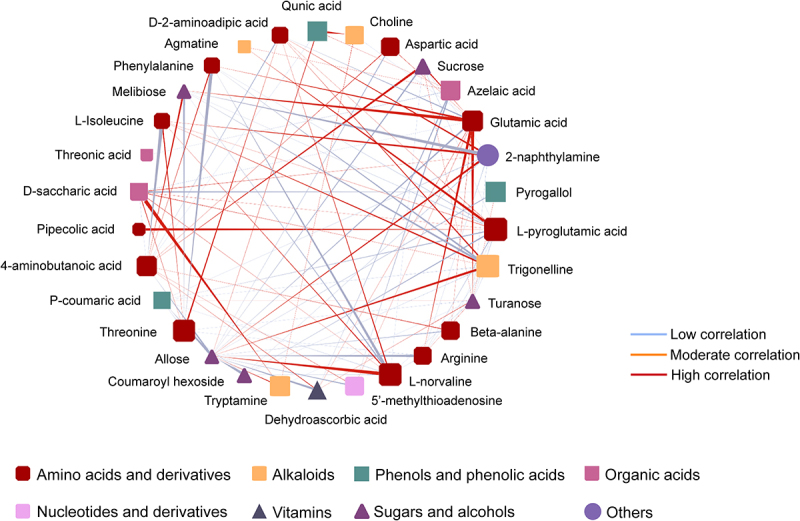
Differential metabolite correlation network. Nodes with different shapes represent different categories of metabolites. The different colors of the lines represent the degree of correlation between metabolites.

Network analysis was performed on all the differential metabolites in pepino fruits during the three developmental stages. A correlation coefficient threshold was set at an absolute value of > 0.8 between metabolites and a correlation coefficient *p* value of 0.05. The top 30 features in abundance of all the differential metabolites were displayed in the network graph (Figure 7). In the metabolite correlation network diagram, the size of the node indicates the connectivity of the metabolite with the rest of the metabolites, with the node representing the stronger the connectivity of the metabolite with the other metabolites, and the line between metabolites representing the degree of correlation between the two metabolites being connected, with a red line representing a high correlation and a blue line representing a low correlation. Among the 94 differential metabolites, there are 12 amino acids and derivatives, 5 sugars and alcohols, 4 alkaloids, 3 phenols and phenolic acids, 3 organic acids, 1 nucleotide and derivatives, 1 vitamin and 1 compound classified as other constitute the metabolite relevance network. The network graph showed that amino acids and derivatives have the most connections with other metabolites in this metabolic network, which indicates that amino acids and derivatives play an extremely important role in the metabolism of pepino, influencing the accumulation of sugars, vitamins, phenols and phenolic acids during the growth and development of pepino. This is basically in line with the biosynthetic pathway, where amino acids and their derivatives are the precursors for the synthesis of metabolites in the organism. In addition to amino acids and their derivatives, azelaic acid, D-saccharic acid, quinic acid and pyrogallol are large nodes in this metabolic network and therefore these metabolites are important hubs in the metabolic network.

### Analysis of the correlation between metabolomics and transcriptomics

**Figure 8.**
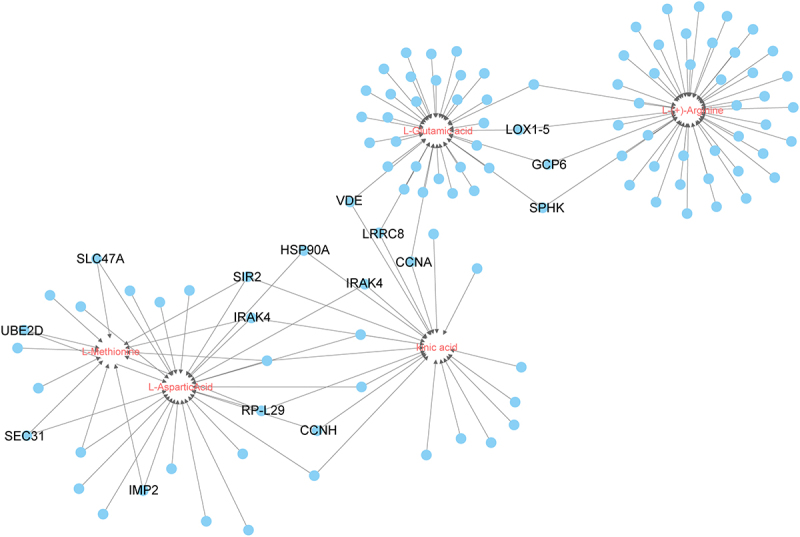
Interaction network of some key DEMs correlated with genes. The blue nodes represent genes, circles in the center of the lines represent metabolites.

Typically, metabolites with VIP scores ≥ 1 are considered to be significantly different. After screening, the selected key metabolites include 4 amino acids: methionine, glutamic acid, aspartic acid, and arginine. Additionally, one phenolic acid, quinic acid, and one organic acid, azelaic acid were identified. We conducted gene mining to explore genes related to the synthesis of these key metabolites in maturation stage pepino fruit (Figure 8). It was found that more genes were associated between methionine and aspartic acid, between aspartic acid and quinic acid, between qunic acid and glutamic acid, and between glutamic acid and arginine, indicating that the corresponding genes are involved in the regulation of multiple. The genes in the central region of the complex were all associated with each other. In the central region, all key node genes were functionally annotated, with the most genes associated between aspartic acid and quinic acid, and the *SIR2*, *IRAK4*, *RP-L29* and *CCNH* genes possibly involved in the modulation of both amino acid and phenolic acid synthesis.

## Discusion

Fruit development and ripening is an intricate physiological and biochemical process encompassing a cascade of physiological and biochemical alterations, encompassing modifications in fruit composition, hormone synthesis, breakdown, pigmentation, and other metabolic transformations.^17^ Studying the process of fruit development is of significant importance as it can unravel the reproductive mechanisms of plants and elucidate the molecular, cellular, and physiological foundations that govern fruit development. Previous studies have paid relatively little attention to the metabolites of pepino, particularly the unreported metabolomic differences during its growth and development process. Our study employed a comprehensive non-targeted metabolomics approach to identify compounds in the ‘Qingcanxiang’ fruit, providing important insights into the metabolomic characteristics and bioactive metabolites during the development stages of pepino. Furthermore, this research serves as a fundamental basis for future extensive studies on pepino metabolites and offers new perspectives for breeding strategies aimed at enhancing fruit quality and efficient nutrient accumulation in pepino.

In this study, a total of 187 metabolites were identified in the ‘Qingcanxiang’ fruit. The majority of these metabolites, including phenolic compounds, flavonoids, and alkaloids, exhibited a gradual accumulation pattern during fruit ripening. The main compounds identified in ‘Qingcanxiang’ showed remarkable similarity to the active compounds found in another Solanaceae crop, eggplant.^9,23^ While many metabolites similar to those found in tomatoes were detected in the ‘Qingcanxiang’ fruit,^24^ specific substances such as lycopene and carotenoids, which are unique to tomatoes, were not detected. The similarity in nutritional composition among pepino, eggplant, and tomato is mainly due to their close relationship as closely related species.^25,26^ From a molecular biology perspective, their genomes share many similarities, leading to commonalities in genetic information and metabolic pathways.^27^

One of the key factors determining the popularity of fruits among consumers is their flavor profile. Acidity, sweetness, and volatile aroma compounds are fundamental sensory attributes that contribute to the overall flavor composition of fruits.^28^ As primary metabolites, organic acids play a regulatory role in the growth and development of plants. They serve as metabolic solutes that help regulate cell osmotic potential and balance excessive cation accumulation. Additionally, organic acids are key components involved in plant responses to nutrient deficiencies, metal ion accumulation, and plant-microbe interactions.^29^ This study identified a total of 34 types of organic acids, which is the highest among all metabolites. Among them, 13 types of organic acids exhibited significant changes during the development of ‘Qingcanxiang’ fruit. The top four organic acids with the highest content in the fruit were citric acid, threonic acid, gluconic acid, and D-(+)-malic acid. Sugar is one of the molecular signals that regulate fruit ripening and senescence, playing a significant role in modulating physiological functions, metabolism, gene expression, as well as providing energy and substrates for plant growth, development, and aging.^30,31^ The main sugar compounds in ‘Qingcanxiang’ fruit include sucrose, turanose, L-sorbose, melibiose, melezitose and maltotriose. Sucrose is the most abundant and predominant sugar in ‘Qingcanxianig’ fruit. The sugar-to-acid ratio is commonly used to assess the ripeness, taste, and overall quality of fruits.^32^ In our research, it was observed that during the developmental stages of pepino fruits from the initial to the growth stage and finally to the maturation stage, there was a gradual decrease in the proportion of organic acids, while the ratio of sugars and alcohols exhibited a continuous increase. Both our study and the study by Sanchez et al.^33^ found that the sucrose content in pepino fruits continuously increased during fruit development, which is consistent with the results of other studies on fruits and vegetables. The increase in the synthesis of primary metabolites, secondary compounds, and sugars during the maturation stage of fruits and vegetables may lead to a decrease in titratable acids.^34–36^ The current hypothesis posits that the observed phenomenon can be attributed to the fruit’s respiration exhibiting a preference for consuming organic acids.^37^ Based on the changes in sugar substances in the ‘Qingcanxiang’ fruit, the accumulation of sugars that determines its sweetness primarily occurs during the stages of fruit growth stage to maturation stage.

In addition to sugars and acids, a multitude of other substances, including phenolic acids, flavonoids, lipids, and alkaloids, exert an influence on and contribute to the determination of fruit flavor, thereby potentially inducing variations in taste.^38–40^ Meanwhile, sugar serves as a crucial precursor material by providing energy and a carbon skeleton for the synthesis of phenolic acid.^10^ Moreover, the identification and composition of amino acids, including glutamic acid, aspartic acid, and lysine, serve as significant parameters for assessing the flavor profile of fruits.^41^ Several studies have verified that serine possess a sweet taste and are classified as the sweet amino acid. While bitter amino acids comprise arginine, L-norvaline, L-isoleucine and tryptophan.^42^ Based on our findings, it is evident that the sweet amino acid serine experiences a downregulation while the bitter amino acid tryptophan undergoes an upregulation. This phenomenon is likely attributed to the maintenance of a relative equilibrium between the two amino acids, thereby producing a taste that is typically neither excessively sweet nor bitter.

There has been a growing interest in amino acid-rich foods as a result of increased understanding of nutritional elements in recent years.^43,44^

The results of our experiments revealed that the fruits of the pepino are rich in a variety of amino acids and their derivatives, a result very similar to that of REDGWELL et al.^45^ on‘El Camino’ pepino. The assessment of the nutritional quality and medicinal properties of foods is significantly influenced by the type and quantity of amino acids present. The essential amino acids, methionine, lysine, tryptophan, threonine, phenylalanine and L-Isoleucine, were identified in this study. The insufficiency of crucial amino acids within the human body may result in the emergence of various ailments. Furthermore, in addition to the aforementioned essential amino acids, the fruits of pepino were found to contain aspartic acid, asparagine, tyrosine, pipecolic acid, L-pyroglutamic acid, histidine, and serine. Notably, aspartic acid, pipecolic acid, and glutamic acid were identified as the top three and most abundant amino acids in the fruits of ‘Qingcanxiang’. These findings are consistent with the research of REDGWELL et al.,^45^ who reported that aspartic acid and glutamic acid constitute 90% of the total free amino acids present in pepino. Amino acids are commonly identified as substances with antioxidant properties.^46^ Aerobic organisms, including humans, possess defense mechanisms to safeguard biomolecules against oxidative damage. Nevertheless, these innate antioxidant defenses are incapable of completely eliminating all reactive oxygen species generated. Consequently, the consumption of antioxidant compounds from dietary sources is necessary to prevent oxidative stress. The requirement for exogenous natural antioxidants is both constant and significant. Aspartic acid, an essential amino acid, plays multiple physiological roles in the growth and development of plants. Similarly, it plays a crucial role in the human body by promoting growth and development, enhancing immunity, supporting liver health, and maintaining cardiovascular health.^47^ Pipecolic acid is a non-protein amino acid that is considered to be an important amino acid involved in plant growth and development.^48^ It can also exert antioxidant, anti-inflammatory, and immune-modulating effects in the human body, thereby aiding in the prevention of autoimmune diseases and cancer, among other health conditions. Studies have found that histidine and glutamate can reduce the generation of hydroxyl radicals, while methionine and lysine exhibit strong antioxidant activity.^49^ In addition, histidine and arginine are also of great significance. Histidine is considered to be an essential amino acid for infants and young children,^50^ but it cannot be synthesized endogenously in sufficient concentrations to meet physiological needs. Therefore, it must be obtained from the diet.^51^ Arginine is a semi-essential amino acid that can function as a secretagogue, stimulating the release of growth hormone and insulin, among others.^52^ There are reports indicating that pepino is recommended for diabetes patients.^53^ It is suggested that these beneficial effects may be attributed to the presence of aforementioned substances, suggesting that pepino possesses high antioxidative, nutritional, and medicinal value, with great potential to develop into a functional agricultural product. Our research has found that amino acids, as essential components in the nutrition of fruits and vegetables, play a crucial role. During the growth and maturation of ‘Qingcanxiang’ fruit, the majority of amino acids and derivatives continuously accumulate, with the stage from fruit growth to maturation being the key accumulation stage.

Pepino contains not only amino acids and derivatives but also phenols, phenolic acids, and flavonoids, which exhibit antioxidant properties. Among these, quinic acid, caffeic acid, and epigallocatechin are the phenolic acids with the highest VIP values. Phenolic compounds, which are a vast group of plant secondary metabolites, are also present.^39^ The naturally occurring antioxidants and free radical scavengers found in fruit phenolics are well-established, and the pharmaceutical properties of these phenolics have been observed in numerous fruit varieties.^54^ Phenolic compounds have garnered the attention of researchers in recent years due to their potential as potent antioxidants capable of safeguarding the body against free radicals.^55^ The findings of these studies collectively demonstrate that pepino fruits harbor a diverse array of antioxidant compounds, thus indicating their significant potential as natural antioxidants.

In terms of nutrient accumulation and flavor development, the growth and maturation stages are deemed critical in the attainment of high-quality pepino fruit. The enhancement of organoleptic quality through sugar-induced taste variations is of utmost significance, as it caters to the diverse preferences of consumers with regards to sweetness.^10^ Hence, it is imperative to allocate attention toward the suitable application of irrigation and fertilization throughout the different stages of fruit development, particularly during the crucial stage of nutrient accumulation. Different soil and water-fertilizer conditions can affect the quality of fruits and the accumulation of carbohydrates.^56–59^ Additionally, the *SIR2*, *IRAK4*, *RP-L29*, and *CCNH* genes were identified as pivotal players in the regulation of amino acid and phenolic acid synthesis in our study. However, further investigation is required to elucidate the specific regulatory mechanisms and interconnections among these genes. As an emerging crop, the enhancement of quality and breeding of various strains of this fruit is poised to become a prominent area of future research. Currently, the utilization of gene editing, genomics, machine learning, and other research methodologies can facilitate more comprehensive and extensive investigations into other aspects of this novel fruit’s development and research. Researches have demonstrated that diverse cultivars of eggplant display disparities in the profiles and concentrations of metabolites.^23^ As such, additional research is warranted to elucidate the constituent composition and quantitative content of metabolites in fruits of other pepino cultivars. Different cultivars of pepinos exhibit physiological and qualitative variations, indicating the necessity and potential for exploring other varieties of pepinos.

## Conclusion

In summary, this study utilized UPLC-QTOF-MS and RNA-seq transcriptome sequencing to analyze the accumulation of metabolites and the molecular regulatory mechanisms involved in important metabolites during different developmental stages of ‘Qingcanxiang’ pepino fruits. A total of 187 metabolites were identified, including amino acids and derivatives, sugars and alcohols, organic acids, and other substances belonging to 12 categories. The cumulative changes of these metabolites during fruit growth were also analyzed. The results revealed that 94 of all the metabolites identified were significantly different, with most of the nutrients accumulating between the growth and maturation stages of the fruit, which were significantly up-regulated, thus determining the critical periods in the development of pepino, namely the growth and maturation stages. Significant variations in amino acids and their derivatives, organic acids, sugars and alcohol are observed in fruit during three different developmental stages. These substances are closely associated with the nutritional quality and flavor profile of pepino. The integration of RNA-seq and metabolomics data revealed that the *SIR2*, *IRAK4*, *RP-L29*, and *CCNH* genes play a crucial role in regulating amino acid and phenolic acid synthesis in pepino. The findings of this investigation offer an additional point of reference for research on the growth, development, and quality of pepino.

## Supplementary Material

Supplemental Material

## Data Availability

Data is contained within the article or supplementary material.

## Data statement

The datasets are publicly available at NCBI with Sequence Read Archive (SRA) accession: PRJNA869347.
